# Merozoite surface protein 3.3C-specific antibodies block the intraerythrocytic development of *Plasmodium falciparum* and induce parasite apoptosis

**DOI:** 10.1186/1475-2875-11-S1-P41

**Published:** 2012-10-15

**Authors:** Lvnne M Harris, Graeme Cowan, Kelwalin Dhanasarnsombut, David Cavanagh

**Affiliations:** 1 Institute of Immunology and Infection Research, University of Edinburgh, Ashworth Laboratories, Kings Buildings, Edinburgh, EH9 3JT, UK

## Background

Antibodies mediate naturally acquired immunity to the asexual blood stages of human malaria. Merozoite surface antigen-specific antibodies inhibit the *in vitro* growth and development of the parasite *P. falciparum*, although the functional mechanisms of this inhibition are not fully understood. In this study, we investigated the functional mechanisms of *in vitro* growth inhibition by antibodies to merozoite surface protein 3.3C.

## Materials and methods

Antibodies were raised by immunization with a recombinant antigen derived from the C-terminal region of merozoite surface protein 3.3 (MSP3.3C). *P. falciparum* blood stage parasites were cultured in the presence of α-apical membrane antigen 1 specific, α-MSP3.3C specific or naïve rabbit IgG. Parasite DNA content and morphology, antibody localisation and the presence of apoptotic markers were monitored over the parasite life cycle by microscopy, flow cytometry and indirect immunofluorescence antibody tests.

## Results

MSP3.3C-specific Immunoglobulin G (IgG) displayed unusual and highly potent anti-parasite properties in growth inhibition assays. This activity appears to be caused by inhibition of the intraerythrocytic development of the parasite and not by inhibition of merozoite invasion. Notably, we have shown that antibodies to MSP3.3C can access the intraerythrocytic parasite post merozoite invasion and effectively block further development of the parasite within the host erythrocyte. Our data indicate that specific IgG to MSP3.3C can prevent the export of MSP3.3 through the parasitophorous vacuole membrane into the erythrocyte cytoplasm. In addition, anti-MSP3.3C antibodies induce several characteristic features of programmed cell death within the parasite.

## Conclusions

The mode of action of MSP3.3C-specific antibodies is to gain access to the intraerythrocytic parasite post invasion and arrest parasite development. This research furthers our understanding of the functional mechanisms underpinning *in vitro* growth inhibition by merozoite surface antigen-specific antibodies and highlights the potential of MSP3.3 as a blood stage vaccine candidate.

